# Machiavellianism in Healthcare: A Literature Review

**DOI:** 10.3390/healthcare14050556

**Published:** 2026-02-24

**Authors:** Maria Kapritsou, Vasiliki Papanikolaou, Nikolaos Maniadakis, Tina Garani-Papadatos, Daphne Kaitelidou, Michalis Mantzanas, Theodoros N. Sergentanis

**Affiliations:** 1 Hellenic Anticancer Institute, “Saint Savvas” Hospital, 11522 Athens, Greece; 2 Department of Public Health Policy, University of West Attica, 11521 Athens, Greece; 3 Center for Health Services Management and Evaluation, Faculty of Nursing, National and Kapodistrian University of Athens, 11527 Athens, Greece; 4 General Hospital of Nea Ionia “Konstantopouleio-Patision”, 14233 Athens, Greece

**Keywords:** Machiavellian, Machiavellianism, healthcare professionals, leadership, hospital, nurse, doctor

## Abstract

**Background/Objectives:** Machiavellianism has long been associated with unethical tendencies and behaviors. High-Mach people have been stereotyped to choose business-related professions, contrary to low-Mach individuals choosing the helping professions. There has been a clear shift in scholarly focus, such as Machiavellian leadership and Machiavellian personality traits in healthcare. The objective of this narrative literature review was to provide a structured synthesis of empirical evidence on Machiavellianism within healthcare settings, focusing on its prevalence, manifestations, and organizational implications, while identifying conceptual and research gaps in the field. **Methods:** Literature research was conducted for articles published in PubMed, Scopus, and Google Scholar from 2014 until 2025. Articles written in English, examining Machiavellian traits in healthcare workers and students, were included in this review. **Results:** The search strategy produced 347 items, of which 11 original studies were included. Machiavellianism was described as a personality trait featuring emotional coldness and manipulativeness for the achievement of one’s own ends; individuals exhibiting those traits may prioritize personal gain over collective welfare. Machiavellian tendencies manifesting in organizational culture often lead to a toxic work environment where manipulation might become normalized. Machiavellians show high commitment to their careers, but low commitment to their current organizations, supervisors, and teams. **Conclusions:** Machiavellianism emerges as a relevant but underexplored personality trait in healthcare, associated with unethical behaviors, reduced organizational commitment, and toxic work environments. Addressing its impact requires ethical leadership development, supportive organizational environments, and early identification during professional training to safeguard workforce well-being and patient safety.

## 1. Introduction

Machiavellianism is one of the Dark Triad personality traits, the latter also involving narcissism and psychopathy. This constellation of traits is often associated with interpersonal hostility and a general lack of regard for others’ needs [1]. Recently, the term “Dark Tetrad personality traits” has been used, enriching the previously known “Dark Triad” with additional sadistic behaviors [2].

Machiavellianism is characterized by high levels of antagonism-related traits, which account for the manipulative and interpersonally exploitative aspects [3]. Machiavellian individuals are thought to be able to delay gratification for long-term goals and keep a strategic focus. Machiavellianism has frequently been associated with a greater likelihood of unethical or strategically manipulative behaviors, although such tendencies may vary depending on contextual and organizational factors. Notably, Machiavellianism in leadership, characterized by manipulative strategies, has emerged as a critical area of investigation; reports have indicated that leaders exhibiting Machiavellian traits might contribute to detrimental organizational cultures, ultimately impacting employee morale and patient care outcomes [4]. Accordingly, Machiavellianism in organizations has been associated with unethical behavior in the workplace, as well as with negative workplace outcomes such as dissatisfaction, stress and emotional exhaustion [5].

Most people appear to view the attributes associated with Machiavellianism as inappropriate within the healthcare sector. Given that patient care is considered a priority, the public often resists the concept of healthcare organizations being run like profit-driven businesses; this perception might set the tone for the conceptual debate that usually exists between healthcare managers and providers of patient care [6]. Patient-care providers prioritize the quality of care to the patient, while case managers are concerned about business aspects.

Nevertheless, the relationship between Machiavellianism and organizational performance remains complex. Some researchers contend that Machiavellian leaders may achieve results through strategic cunning, yet these gains often come at the expense of ethical standards and employee well-being [7,8]. This tension between strategic effectiveness and ethical integrity presents a significant challenge for healthcare institutions striving to balance performance with moral responsibility. Collectively, these themes underscore the multifaceted implications of Machiavellianism that are critical to understanding its pervasive influence in healthcare settings [9].

To the best of our knowledge, no prior review has specifically synthesized empirical evidence on Machiavellianism in healthcare contexts. Therefore, the primary objective of this narrative literature review is to provide a structured and critical synthesis of empirical studies focusing specifically on Machiavellianism within healthcare settings. Other Dark Triad or Dark Tetrad traits are discussed only insofar as they offer conceptual or comparative context. The review further seeks to identify recurring patterns, conceptual inconsistencies, and research gaps to inform future research and organizational practice.

## 2. Methods

This study was conducted as a narrative literature review aiming to identify and synthesize relevant empirical studies rather than to achieve systematic exhaustiveness. A broad search strategy was employed to capture key publications on Machiavellianism within healthcare contexts. Electronic databases including PubMed and Scopus were searched, complemented by Google Scholar to identify additional relevant studies, gray literature, and articles that may not be indexed uniformly across databases. The search covered publications from 2014 to 2025 and used combinations of keywords related to Machiavellianism and healthcare professions. The purpose of the search strategy was to identify conceptually and empirically relevant studies rather than to follow a formal systematic review protocol.

**Figure 1:**
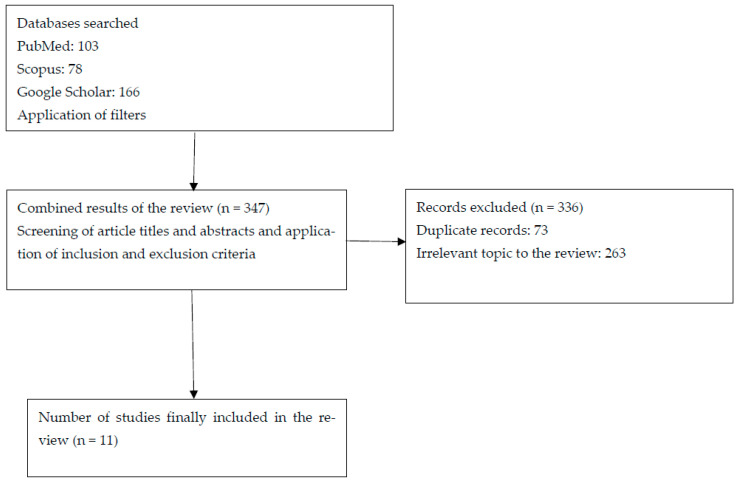
Flow diagram of study selection.

Studies written in English, on healthcare workers, students or managers, were included. Data extraction was performed using a pre-specified template that included the following: first author, year of publication, country where the study was performed, aim of the study regarding Machiavellianism, study design and sample size, method and tools used for the conceptualization of Machiavellianism, and main results. Identification and data extraction were performed by two reviewers (MK and GK) (Figure 1). Given the narrative design of the review and the heterogeneity of study designs and measurement instruments, no formal risk-of-bias or quality assessment tool was applied; emphasis was placed on conceptual relevance and empirical contribution. Although several included studies examined Machiavellianism within the broader framework of the Dark Triad or Dark Tetrad, only findings specifically related to Machiavellianism were extracted and synthesized for the purposes of this review.

Machiavellianism was identified in the included studies through validated psychometric instruments as defined by the original authors (e.g., MACH-IV, Dirty Dozen, Short Dark Triad, or related validated scales). The present review did not apply independent diagnostic criteria but relied on the operational definitions and measurement approaches adopted in each primary study.

The search process identified 347 records. Following screening of titles and abstracts, 336 records were excluded because they were unrelated to healthcare populations, did not assess Machiavellianism, or were theoretical, editorial, or review articles. Eleven full-text articles met the predefined inclusion criteria and were considered relevant for thematic synthesis. All eleven studies were included in the qualitative synthesis, as they examined Machiavellian traits among healthcare professionals, students, or managers and were published in English. Data extraction was independently performed by two reviewers using a standardized template, ensuring consistency and methodological rigor. The final sample comprised cross-sectional and observational studies conducted across diverse healthcare systems, providing a comprehensive overview of Machiavellianism in healthcare organizations. Given the diversity of study designs, populations, and measurement tools, heterogeneity across findings was anticipated and is addressed in the interpretation of results.

## 3. Results

The results of this review synthesize findings from eleven empirical (i.e., original quantitative or observational) studies examining Machiavellianism within healthcare education and practice. Rather than presenting results on a study-by-study basis, the evidence is organized thematically to highlight recurring patterns across populations, career stages, and organizational contexts. The included studies encompass healthcare students, trainees, and professionals from diverse cultural and institutional settings, using a range of validated measures of Machiavellianism and related constructs. Collectively, the findings illustrate how Machiavellian traits are distributed across healthcare populations, how they manifest during education and early career development, and how organizational environments shape their behavioral expression. Eleven studies were included in this literature review; their characteristics and main findings are summarized in Table 1. Themes were derived through iterative reading of the included studies and inductive identification of recurring conceptual patterns related to levels and distribution of Machiavellianism, manifestations during education and early career stages, and organizational consequences. This thematic organization reflects a narrative synthesis approach rather than a predefined analytical framework.

### 3.1. Levels and Distribution of Machiavellianism in Healthcare Populations

This section synthesizes evidence on the prevalence and distribution of Machiavellianism across healthcare students and professionals. The included studies indicate that Machiavellian traits are present within healthcare populations, albeit with variability across career stages, professional roles, and demographic characteristics. The studies report moderate to relatively high levels of Machiavellianism among medical students and early-career professionals, while others indicate lower overall levels compared to the general population. Gender differences emerge as a recurrent finding, with males typically scoring higher than females. Collectively, these findings challenge the assumption that healthcare professions are uniformly characterized by prosocial personality profiles and underscore the importance of examining individual differences within helping professions. However, variability across findings should also be interpreted in light of differences in study designs, measurement instruments, and sample characteristics.

Bratek et al. [10] explored Machiavellian levels on 509 respondents (16.1% medical school candidates, 65% medical students, 9.8% medical trainees, 6.3% residents, and 2.8% specialists) through the Mach-IV score self-report questionnaire. The overall mean Mach-IV score was 59.24 (SD = 6.07). The highest mean Mach-IV score, 61.80, was reported with the group of medical students and the lowest mean Mach-IV score, 57.61 (7.88) was reported with the registered specialist group. Gender differences remained statistically significant, where 47.33% of women and 60.18% of men were “high Machs” (*p* = 0.016). Two general subgroups, “low Machs” (<60 points) and “high Machs” (≥60 points), turned out to be approximately equal in number: 49.5% and 50.5%, respectively. The Machiavellianism levels among medical candidates, students, and doctors were relatively high; however, they were gradually decreasing with the progress of career (*p* = 0.0196) [10].

Bucknall et al. [11] noted that, in comparison with the general population, healthcare professionals scored significantly lower on the rate of narcissism, Machiavellianism, and psychopathy (12.0, 53.0, and 44.7 on average) (*p* < 0.001). Among medical professionals, surgeons remained in the top 3 for Machiavellianism. All healthcare workers scored low on the Dark Triad; therefore, the authors supported that the stereotype of healthcare staff as untrustworthy and greedy does not apply in general, even when considering only surgeons [11].

On the other hand, Braithwaite et al. [12] also reported a significant difference in Machiavellianism scores between women with 6–10 years of work experience (mean = 3.15, SD = 0.097) and women with more than 20 years of work experience (mean = 3.47, SD = 0.06); mean difference = 0.325, *p* = 0.044, 95% CI: 0.005 to 0.646. Similarly, there was a significant difference in Machiavellianism scores between men with <5 years of work experience (mean = 2.23, SD = 0.21) and men with >20 years of work experience (mean = 3.20, SD = 0.12); mean difference = 0.968, *p* = 0.006, 95% CI: 0.209 to 1.726. These findings indicate that Machiavellianism scores tended to increase with years of professional experience in both male and female participants, although the magnitude of differences varied across gender and career stages [12].

The study of Bujok et al. [13] examined a cohort of 380 first-year and 217 third-year medical students in Germany. Participants completed the Dirty Dozen (DD) and Multi-Motive Grid (MMG) questionnaires. The analysis explored differences in the Dark Triad personality traits—narcissism, psychopathy, and Machiavellianism—between medical students and a reference sample, as well as between the two cohorts of students. The findings indicated no statistically significant differences between first-year and third-year medical students in terms of the Dark Triad traits. Similarly, no remarkable differences were observed between the medical student group and the reference sample (n = 501), except for higher psychopathy scores among the former. Gender analysis revealed that male students scored significantly higher on all three Dark Triad traits compared to their female counterparts. Regarding implicit motives measured by the MMG, first-year students demonstrated significantly higher levels of Fear of Rejection but recorded lower levels of Hope of Success and Hope of Power compared to third-year students. The results suggest that Dark Triad traits are present prior to the commencement of medical studies and do not significantly differ between medical students and non-medical reference samples [13].

Overall, the included studies converge in indicating that Machiavellian traits are present across healthcare populations, although the magnitude and direction of associations vary depending on gender, career stage, and measurement approach.

### 3.2. Manifestations of Machiavellianism During Education and Early Career Stages

This section focuses on how Machiavellian traits manifest during medical education and early professional development. Evidence suggests that Machiavellianism can be detected early, often prior to or at the beginning of formal training, and may remain relatively stable throughout medical education. The studies link higher Machiavellianism to maladaptive behaviors, particularly academic misconduct, highlighting how strategic, manipulative tendencies may emerge in competitive educational environments. Additionally, the role of ethics education and institutional norms appears critical in shaping the expression of these traits. Together, these findings indicate that medical training represents a key developmental period during which Machiavellian tendencies may either be reinforced or mitigated.

A total of 591 medical students enrolled in their first, third, and fifth years at a Portuguese medical school participated in a study employing the Dark Triad Dirty Dozen, Ryff’s Psychological Well-Being Scales, and a customized Academic Misconduct Questionnaire within a cross-sectional framework. The findings revealed that fifth-year medical students with elevated levels of Machiavellianism and psychological well-being, who also perceived higher incidences of peer fraud and lesser consequences for cheating, reported greater engagement in academic misconduct. The proposed model explained 16.6% of the variance in academic misconduct. Among the variables, Machiavellianism emerged as the strongest predictor of cheating behavior, whereas sex and age were not statistically significant factors. This study provides valuable insights into the role maladaptive personality traits play in fostering academic dishonesty among medical students, while also highlighting the influence of psychological and contextual elements. These results can serve as a foundation for developing institutional strategies aimed at promoting academic integrity within the training of future healthcare professionals. Although this study does not directly examine leadership, its findings are relevant to leadership research, as Machiavellian traits identified in medical students may represent early antecedents of unethical or manipulative leadership behaviors later in professional life. The association between Machiavellianism and academic misconduct highlights how maladaptive personality traits can manifest before individuals assume formal leadership roles. These findings underscore the importance of early educational and organizational interventions aimed at promoting ethical conduct and leadership development in healthcare [14].

Constantin et al. [15] examined the attitudes of Romanian medical students and doctors toward business ethics, focusing on preferences for five ethical philosophies: Machiavellianism, moral objectivism, social Darwinism, ethical relativism, and legalism, as well as the influence of sex, age, and ethics education. The sample consisted of 53 medical students, 192 doctors, and 108 management students (total N = 353), with mean ages of 24.4, 31.5, and 20.8 years, respectively. Participants completed the Attitudes Toward Business Ethics Questionnaire (ATBEQ), which demonstrated good reliability (α = 0.807). Results showed that moral objectivism was the dominant ethical philosophy among both medical students (mean = 3.21, SD = 0.63) and doctors (mean = 3.25, SD = 0.52), significantly exceeding Machiavellianism, ethical relativism, social Darwinism, and legalism (students: F(4.208) = 22.88, *p* < 0.001; doctors: F(4.764) = 64.53, *p* < 0.001). Medical students reported significantly lower Machiavellianism than management students (F(1.159) = 56.69, *p* < 0.001). No significant differences were found between medical students and doctors for most dimensions, except legalism (F(1.256) = 4.51, *p* = 0.035). Regression analyses indicated that ethics education significantly predicted lower Machiavellianism (beta = −0.57, *p* < 0.001), social Darwinism (B = −0.26, *p* < 0.001), ethical relativism, and legalism, while age showed no significant effects. The study concluded that ethics education plays a central role in shaping ethical attitudes toward business in medical contexts [15].

Collectively, these findings suggest that Machiavellian tendencies may emerge early in professional formation and interact with educational environments, with ethical training appearing as a potential moderating factor.

### 3.3. Organizational Context, Workplace Conditions, and Behavioral Consequences

This section examines the interaction between Machiavellianism and organizational contexts within healthcare settings. The reviewed studies indicate that Machiavellian traits are strongly associated with counterproductive work behaviors, reduced affective commitment, and strained interpersonal relationships, particularly in environments characterized by perceived injustice, weak accountability, or psychological contract breaches. Importantly, organizational factors frequently act as mediators or moderators, shaping how Machiavellian tendencies are expressed in practice. These findings support the view that Machiavellianism is not solely an individual-level risk factor but one that is highly sensitive to workplace conditions. In healthcare organizations, such dynamics may have significant implications for teamwork, staff well-being, and quality of care.

The aim of Ying and Cohen [16] was to examine the relationship between Dark Triad Personality (DTP) traits—psychopathy, narcissism, and Machiavellianism—and counterproductive work behaviors (CWBs) among physicians in China, with emphasis on the mediating role of organizational factors. Data were collected from 168 physicians (response rate 84%) at Beijing Children’s Hospital using validated self-report questionnaires rated on a 7-point Likert scale. Regression and mediation analyses with 1000 bootstrapped samples were conducted [16]. Results showed a significant positive association between the overall DTP score and both interpersonal (CWBI) and organizational CWBs (CWBO) (β = 0.27–0.33, *p* < 0.001). Among individual traits, Machiavellianism demonstrated the strongest and most consistent effects on CWBI (β = 0.33, *p* < 0.001) and CWBO (β = 0.45, *p* < 0.001), while narcissism and psychopathy were largely non-significant. Organizational justice and organizational commitment emerged as key mediators, particularly for CWBI and CWBO respectively, whereas psychological contract breach showed no mediating effect. Accountability showed partial mediation effects [16].

Li et al. [2] sought to examine the effects of Dark Tetrad personality traits (narcissism, psychopathy, Machiavellianism, and everyday sadism) on counterproductive work behavior (CWB) among doctors in Pakistan, as well as the mediating role of psychological contract breach (PCB) and the moderating effect of political skills. Data were collected from 400 doctors working in public sector hospitals in Punjab, Pakistan (response rate 83.6%; 54.9% male). Participants completed validated self-report measures, including the Short Dark Triad (SD3), the Short Sadistic Impulse Scale (SSIS), a psychological contract breach scale, a political skills scale, and an 8-item CWB scale. Structural equation modeling using PLS-SEM revealed that all Dark Tetrad traits had significant positive direct effects on CWB: narcissism (β = 0.144, *p* = 0.005), psychopathy (β = 0.207, *p* < 0.001), Machiavellianism (β = 0.132, *p* = 0.016), and sadism (β = 0.251, *p* < 0.001). Psychological contract breach also significantly predicted CWB (β = 0.267, *p* < 0.001) and partially mediated the relationships between all Dark Tetrad traits and CWB (variance accounted for, range: 13.5–26.7%). The structural model explained 20.8% of the variance in PCB and 34.9% of the variance in CWB (standardized root mean square, SRMR = 0.058). Political skills significantly moderated the relationships between narcissism and PCB (β = 0.126, *p* = 0.021) and psychopathy and PCB (β = 0.125, *p* = 0.026), but not those involving Machiavellianism or sadism. The study concludes that dark personality traits are important predictors of counterproductive behavior in healthcare settings, particularly when psychological contracts are perceived as breached [2].

In a separate study conducted during the COVID-19 pandemic, Shengbo et al. [17] examined the relationship between Machiavellianism and anxiety. Results of the measurement model demonstrated that the scales were reliable and valid. Loneliness was examined as a mediator between Machiavellianism and anxiety; however, it did not have a significant role in this relationship. The results indicated b = −0.04, t = −0.70, *p* > 0.05, with a 95% CI ranging from −0.14 to 0.06. Therefore, no significant indirect effect of loneliness was found between Machiavellianism and anxiety [17].

Kaufmann et al. [18] investigated two forms of precarious employment—career interruptions and part-time or casual work—as potential moderators in the relationship between the dark triad personality traits (Machiavellianism, narcissism, and psychopathy) and professional commitment. The research sample comprised 184 Australian professionals, of whom 52.2% were men. A significant proportion of participants reported experiences of career interruptions (69.6%) or a year or more of part-time or casual employment (70.7%). The findings revealed that psychopathy was negatively associated with affective commitment, while Machiavellianism demonstrated a positive relationship with normative commitment. Narcissism, on the other hand, exhibited positive correlations with both normative and continuance commitment. Through regression analysis, it was observed that individuals with longer durations of part-time or casual employment exhibited stronger negative associations between Machiavellianism and psychopathy with affective commitment. Conversely, among those who experienced considerable career interruptions, Machiavellianism displayed a stronger positive association with continuance commitment. These insights contribute to a deeper understanding of the dark triad’s nuanced influence on workers’ professional attachment within the context of precarious employment [18].

Although the study examined the Dark Tetrad traits collectively, the present review focuses specifically on the findings related to Machiavellianism. Moraes et al. [19] explored the association between substance abuse and Dark Tetrad personality traits (psychopathy, narcissism, Machiavellianism, and everyday sadism) among health sciences and non-health sciences students. The sample consisted of 174 Brazilian university students aged 18–58 years (M = 25.60, SD = 9.14), of whom 82.75% were women. Participants completed validated self-report instruments assessing psychopathy (LSRP), narcissism (PNI), Machiavellianism (FFMI), everyday sadism (SSIS), and substance use (ASSIST). Correlation analyses revealed significant positive associations between secondary psychopathy and the use of tobacco, alcohol, cannabis, and hallucinogens (*p* < 0.001). Vulnerable narcissism was positively associated with hallucinogen use, while everyday sadism showed a positive relationship with alcohol consumption. In contrast, Machiavellianism demonstrated weak or negative associations with substance use. MANOVA results showed no main effect of sex on substance use (*p* = 0.412) and no sex-by-field interaction (*p* = 0.754). A significant but small effect of field of study was observed (η^2^ = 0.062), with non-health sciences students consuming more hallucinogens than health sciences students (*p* = 0.020; d = 0.49). Regarding personality traits, men scored significantly higher than women on primary psychopathy, Machiavellian antagonism, and planfulness (η^2^ = 0.150). Notably, planfulness was conceptualized within the Machiavellianism framework as a strategic and goal-oriented tendency. In this context, it reflects calculated and instrumental behavior that may serve self-interest rather than prosocial or cooperative aims. The findings suggest that dark personality traits, particularly psychopathy and sadism, are linked to risky substance use behaviors, highlighting potential implications for students’ future professional practice. These findings suggest that, within this broader personality framework, Machiavellianism appears less strongly associated with substance use compared to psychopathy and sadism [19].

Across organizational contexts, Machiavellianism consistently appears linked to counterproductive behaviors, yet its expression is shaped by workplace climate, perceived justice, and structural constraints.

## 4. Discussion

This literature review provides a structured synthesis of empirical evidence published between 2014 and 2025 on Machiavellianism within healthcare settings, highlighting its prevalence, manifestations, and organizational consequences among healthcare professionals and students. Across the included studies, Machiavellianism consistently emerged as a salient personality trait influencing unethical behavior, counterproductive work behaviors [10], academic misconduct [13], and diminished organizational commitment [10], underscoring its relevance in healthcare organizations that are traditionally grounded in altruism and ethical responsibility.

A recurrent finding across studies was that Machiavellianism is not absent from healthcare populations, despite common assumptions that helping professions attract predominantly prosocial individuals. Several studies reported moderate to relatively high Machiavellian scores among medical students and professionals, with evidence suggesting that such traits might already be present prior to professional socialization and persist throughout career progression [10,13]. This observation aligns with broader literature suggesting that certain Dark Triad traits, including narcissism and Machiavellianism, may be attracted to professions associated with prestige, authority, and decision-making power, although the healthcare context introduces strong normative and ethical counterbalances. Importantly, some findings indicated that Machiavellian tendencies might decrease with advancing career stage, potentially reflecting professional norms, ethical training, or institutional constraints [10]. However, other studies demonstrated stability or even increases in Machiavellian traits with experience, particularly among males, suggesting heterogeneous developmental trajectories influenced by gender, role expectations, and organizational pressures [12].

The review also revealed robust associations between Machiavellianism and counterproductive or unethical behaviors in both educational [14] and clinical contexts [14]. Machiavellianism was consistently identified as a strong predictor of academic misconduct among medical students, outperforming narcissism and psychopathy in explanatory power [14]. These findings could raise concerns regarding the ethical formation of future healthcare professionals, as early manifestations of manipulative and self-serving behavior may carry over into clinical practice. Similarly, studies conducted among practicing physicians demonstrated that Machiavellianism is positively associated with counterproductive work behaviors, including interpersonal deviance and organizational misconduct [2,16]. This convergence of evidence across career stages reinforces the notion that Machiavellianism may constitute a persistent risk factor within healthcare systems.

Organizational context emerged as a critical moderator shaping the expression of Machiavellian traits. Multiple studies emphasized that Machiavellian individuals are particularly responsive to situational cues, such as perceived injustice, weak accountability structures, and toxic leadership climates [16,18]. These findings align with theoretical perspectives suggesting that Machiavellians strategically adapt their behavior to environmental constraints and opportunities. In healthcare organizations characterized by high stress, hierarchical structures, and resource scarcity, such contextual vulnerabilities may inadvertently legitimize manipulative strategies and erode ethical standards.

Another important theme concerns commitment and professional attachment. Evidence indicated that Machiavellian individuals tend to demonstrate strong commitment to their careers but weaker affective commitment to their organizations, supervisors, or teams [18]. In healthcare settings, this pattern may translate into prioritization of personal advancement over collective goals, potentially undermining teamwork, trust, and continuity of care. This distinction between career commitment and organizational commitment is particularly relevant in healthcare, where collaborative practice is essential for patient safety and quality outcomes.

Gender differences were also recurrently reported, with males exhibiting higher Machiavellian scores than females across student and professional samples [10,13,19]. These findings may reflect broader socialization patterns or differential reinforcement of competitive and instrumental behaviors. However, the literature remains limited in explaining the mechanisms underlying these differences, highlighting the need for more nuanced, gender-sensitive analyses.

Finally, while some authors suggested that Machiavellian traits may confer short-term strategic advantages, particularly in high-pressure or leadership roles, the overarching evidence points toward predominantly negative organizational consequences. Toxic work environments, reduced trust, emotional exhaustion, and increased turnover intentions are repeatedly linked to Machiavellian leadership and behaviors [8,20]. In healthcare, such outcomes pose serious risks not only to staff well-being but also to patient care quality and institutional credibility.

Despite the growing empirical interest, important research gaps remain. The predominance of cross-sectional designs limits causal inference and prevents understanding of developmental trajectories of Machiavellian traits across medical training and professional life. Future research should incorporate longitudinal designs to clarify stability and change over time. Moreover, the current literature is heavily concentrated in specific geographical contexts, particularly Europe and Asia, limiting cross-cultural comparisons. Comparative international studies would enhance understanding of how institutional norms and cultural factors shape the expression of Machiavellian traits in healthcare. Additionally, qualitative and mixed-methods research remains scarce. In-depth qualitative investigations could provide valuable insight into how Machiavellian behaviors are perceived, rationalized, and experienced within healthcare teams, thereby enriching the predominantly quantitative evidence base.

## 5. Study Limitations

This review is subject to several limitations. First, the majority of included studies employed cross-sectional designs, limiting causal inference. Second, heavy reliance on self-report measures raises concerns regarding social desirability and response bias, particularly when assessing unethical or counterproductive behaviors. Third, substantial heterogeneity existed across studies in terms of instruments, samples, cultural contexts, outcome variables, and inclusion criteria, which restricts direct comparability and may partly explain variability across findings. Furthermore, several psychometric instruments were developed and validated in specific cultural contexts and were applied in diverse healthcare systems. Cross-cultural differences in the interpretation of personality constructs may influence measurement equivalence and should be considered in future research. Additionally, most studies were conducted in specific national contexts, limiting generalizability across healthcare systems. Finally, longitudinal and qualitative studies remain scarce, constraining insight into developmental processes and lived experiences.

## 6. Conclusions

In conclusion, this review demonstrates that Machiavellianism represents a salient and consequential personality trait within healthcare education and practice. Evidence consistently links Machiavellian tendencies to unethical behavior, counterproductive work behaviors, reduced organizational commitment, and toxic workplace dynamics. While contextual factors can amplify or attenuate these effects, the cumulative findings highlight the importance of addressing both individual traits and organizational environments. Healthcare institutions should prioritize ethical leadership development, fair organizational practices, and early identification of maladaptive traits during training. Although the number of eligible studies remains relatively limited, the available evidence highlights consistent patterns that warrant further investigation. Future research should prioritize longitudinal and mixed-method designs, expand cross-cultural comparisons, and incorporate qualitative approaches to better understand the mechanisms, contextual moderators, and developmental trajectories of Machiavellianism in healthcare settings.

## Figures and Tables

**Table 1:** Description of methods and results in the included studies.

Study	Aim	Study Design and Sample Size	Method/Tools	Results
Li et al. [2] Pakistan	To examine the effects of Dark Tetrad traits on counterproductive work behavior, with PCB as mediator and political skills as moderator	Cross-sectional study design, 400 doctors from public hospitals in Punjab, Pakistan; 54.9% male; response rate 83.6%	SD3 (narcissism, psychopathy, Machiavellianism), SSIS (sadism), PCB scale, Political Skills scale, CWB scale. Analysis: PLS-SEM with 5000 bootstrap samples	All Dark Tetrad traits positively predicted CWB: narcissism β = 0.144 (*p* = 0.005), psychopathy β = 0.207 (*p* < 0.001), Machiavellianism β = 0.132 (*p* = 0.016), sadism β = 0.251 (*p* < 0.001). PCB predicted CWB (β = 0.267, *p* < 0.001) and partially mediated all relationships (variance accounted for: 13.5–26.7%). Model explained 34.9% of CWB variance; standardized root mean square, SRMR = 0.058
Bratek et al. [10] Poland	To assess the level of Machiavellianism	Prospective descriptive study, N = 509 respondents: medical school candidates (16.1%), medical students (65%), medical trainees (9.8%), residents (6.3%) and specialists (2.8%)	Mach-IV score, a self-report questionnaire	The group of registered specialists exhibited the lowest mean Mach-IV score at 57.61 ± 7.88. A positive correlation was observed between male gender and higher Mach-IV scores, with women demonstrating a mean score of 58.97 ± 6.08, compared to 60.16 ± 6.01 in men. Conversely, age among post-graduate participants was negatively associated with mean Mach-IV scores. Participants were further categorized into two subgroups, namely “low Machs” (scores < 60) and “high Machs” (scores ≥ 60), which were similarly distributed, comprising 49.5% and 50.5% of the sample, respectively. Gender-based differences remained statistically significant; 47.33% of women and 60.18% of men were classified as “high Machs.”
Bucknall et al. [11] United Kingdom	The assessment of the levels of dark triad personality traits (narcissism, Machiavellianism, and psychopathy) among individuals working in health care versus the general population	Cross-sectional study, N = 248 healthcare professionals	The Narcissistic Personality Inventory, MACH-IV scale, The 26-statement Levenson Self-Report Psychopathy Scale	Healthcare professionals demonstrated significantly lower scores in narcissism, Machiavellianism, and psychopathy when compared to the general population (*p* < 0.001). Among the healthcare subgroups, nursing professionals exhibited notably higher levels of secondary psychopathy relative to medical professionals (*p* = 0.04, mean LSRP score of 20.3). Furthermore, within the medical professional cohort, surgeons displayed significantly elevated levels of narcissism (*p* = 0.03, mean NPI score of 15.0).
Braithwaite et al. [12] Australia	To examine the basis of multidisciplinary teamwork and stereotypical behaviors, measuring five personality dimensions, viz openness, conscientiousness, agreeableness, neuroticism and extroversion such as Machiavellian Personality and Social Conservatism	Prospective study, clinical professionals (n = 133) divided into 35 groups of doctors, nurses and allied health professionals, or mixed professions	Three team-based experimental tasks to assess different aspects of intragroup interactions	There was a significant difference in Machiavellianism scores between men with <5 years of work experience (mean = 2.23, SD = 0.21) and men with more than 20 years of work experience (mean = 3.20, SD = 0.12); mean difference = 0.968, *p* = 0.006, 95% CI: 0.209 to 1.726. Similarly, a notable difference was observed in female Machiavellianism scores between individuals with 6 to 10 years of experience (mean = 3.15, SD = 0.097) and those with more than 20 years of experience (mean = 3.47, SD = 0.06). The mean difference was 0.325, with a *p*-value of 0.044 and a 95% CI ranging from 0.005 to 0.646.
Bujok J et al. [13] Germany	To assess Dark Triad traits and implicit motives among medical students, compare cohorts, gender differences, and reference sample	Cross-sectional design. 597 medical students in Germany (380 first-year, 217 third-year; 357 female, 238 male). Comparison was performed with a reference sample (n = 501)	Dirty Dozen (DD) for narcissism, Machiavellianism, psychopathy; Multi-Motive Grid (MMG) for implicit motives. Statistical analyses: MANOVA, ANOVA, *t*-tests, Pearson correlations	No differences in narcissism (*p* = 0.167), psychopathy (*p* = 0.404), or Machiavellianism (*p* = 0.804) were noted between the first-year and third-year cohorts. Male students scored higher on Machiavellianism (mean difference = 0.83, *p* < 0.001). A small effect was noted versus the reference sample only for psychopathy. First-year students had higher Fear of Rejection (*p* = 0.025) and lower Hope of Success (*p* = 0.003) and Hope of Power (*p* = 0.022).
Veríssimo et al., [14] Portugal	Evaluation of associations of academic misconduct with dark personality traits and psychological well-being, as future healthcare worker	Prospective observational study, N = 591 medical students	The Portuguese version of Ryff’s Psychological Well-Being Scales (PWBS) was applied.	The three dark traits demonstrated strong and significant correlations with the DTDD (r > 0.670). Among the inter-trait correlations, the strongest association was between Machiavellianism and psychopathy (r = 0.434, *p* < 0.01). The DTDD (r = 0.238) and its dimensions showed significant positive correlations with academic misconduct, with Machiavellianism exhibiting the highest correlation (r = 0.295), followed by Narcissism (r = 0.113, *p* < 0.01) and psychopathy (r = 0.093, *p* < 0.05). Conversely, psychological well-being was negatively correlated with the DTDD (r = −0.173), including its Machiavellianism (DTDD-M; r = −0.168) and psychopathy (DTDD-P; r = −0.140) dimensions (*p* < 0.01). No significant correlations were found between psychological well-being and academic misconduct (r = 0.052, *p* = 0.233). Perceptions surrounding peer fraud and the severity of penalties for cheating were positively (r = 0.276) and negatively correlated (r = −0.136) with the Academic Misconduct Questionnaire (*p* < 0.01), respectively. No statistically significant relationships were observed between cheating-related perceptions and personality or psychological factors.
Constantin et al. [15] Romania	To assess attitudes toward business ethics and the influence of sex, age, and ethics education among medical students and doctors	Prospective observational study, 53 medical students (mean age = 24.4), 192 doctors (mean age = 31.5), 108 management students (mean age = 20.8); total N = 353	Attitudes Toward Business Ethics Questionnaire (ATBEQ, α = 0.807). Analyses: repeated-measures ANOVA, between-group ANOVA, multiple regression	Moral objectivism was highest for students (mean = 3.21, SD = 0.63) and doctors (mean = 3.25, SD = 0.52). Significant differences across ethical philosophies (students: F = 22.88, *p* < 0.001; doctors: F = 64.53, *p* < 0.001). Medical students scored lower in Machiavellianism than management students (F = 56.69, *p* < 0.001). Ethics education predicted lower Machiavellianism (beta = −0.57, *p* < 0.001). The association between age and Machiavellianism was non-significant.
Ying and Cohen [16] China	To examine the relationship between Dark Triad Personality (psychopathy, narcissism, Machiavellianism) and counterproductive work behaviors (interpersonal and organizational), and to test the mediating role of organizational factors on counterproductive work behaviors (CWBs), based on the argument that situational variables mediate this relationship	Prospective observational study, N = 168 physicians	Cross-sectional survey design. Paper-and-pencil questionnaires using 7-point Likert scales. Instruments: Dark Triad Dirty Dozen (α = 0.87 total), CWB scale (CWBI α = 0.86; CWBO α = 0.67), Organizational Justice (α = 0.96), Organizational Commitment (α = 0.90), Accountability (α = 0.83), Psychological Contract Breach (α = 0.68). Data analysis: regression and multiple mediation with 1000 bootstrapped samples, narcissism (reliability = 0.88), and Machiavellianism (reliability = 0.87).	Dark Triad total significantly predicted CWB-individual and CWB-Organization (β = 0.27–0.33, *p* < 0.001). Machiavellianism showed strong effects on CWBI (β = 0.33, *p* < 0.001) and CWBO (β = 0.45, *p* < 0.001). Narcissism and psychopathy were non-significant in regressions. Organizational justice mediated effects on CWBI, and organizational commitment mediated effects on CWBO. Psychological contract breach showed no mediating effect
Shengbo L et al., [17] China	To explore the mediating role of loneliness, Autonomous Sensory Meridian Response (ASMR), on the relationship between narcissism, Machiavellianism, psychopathy and anxiety disorder	Prospective observational study, N = 512 healthcare professionals and bioinformatics in Beijing	A questionnaire via WeChat	Results of the measurement model demonstrated that the scales were reliable and valid. Loneliness was examined as a mediator between Machiavellianism and anxiety; however, it did not have a significant role in this relationship. Νo significant indirect effect of loneliness was found between Machiavellianism and anxiety.
Kaufmann et al. [18] Australia	The exploration of the relationship between the DT traits (narcissism, Machiavellianism, and psychopathy) and professional commitment of Australian healthcare workers in an undergraduate psychology unit	Prospective observational study, N = 184 employed adult Australians recruited by students enrolled in an undergraduate psychology unit	The 12-item “Dirty Dozen” concise measure of the DT	The results showed a significant interaction between Machiavellianism and years in part-time and casual employment (R^2^ = 0.04, F(1.174) = 8.39, *p* < 0.01), and an interaction between psychopathy and years in part-time and casual employment (R^2^ = 0.03, F(1.174) = 6.08, *p* = 0.02), in their impact on affective commitment.

## Data Availability

The original contributions presented in this study are included in the article. Further inquiries can be directed to the corresponding author.
